# PheSom: a term frequency-based method for measuring human phenotype similarity on the basis of MeSH vocabulary

**DOI:** 10.3389/fgene.2023.1185790

**Published:** 2023-07-11

**Authors:** Xinhua Liu, Ling Gao, Yonglin Peng, Zhonghai Fang, Ju Wang

**Affiliations:** ^1^Department of Biochemistry and Molecular Biology, School of Basic Medical Sciences, Hangzhou Normal University, Hangzhou, Zhejiang, China; ^2^ School of Biomedical Engineering and Technology, Tianjin Medical University, Tianjin, China; ^3^ Shanghai Center for Systems Biomedicine, Shanghai Jiao Tong University, Shanghai, China

**Keywords:** phenotype, mesh, OMIM, similarity score, FIDC

## Abstract

**Background:** Phenotype similarity calculation should be used to help improve drug repurposing. In this study, based on the MeSH terms describing the phenotypes deposited in OMIM, we proposed a method, namely, PheSom (Phenotype Similarity On MeSH), to measure the similarity between phenotypes. PheSom counted the number of overlapping MeSH terms between two phenotypes and then took the weight of every MeSH term within each phenotype into account according to the term frequency-inverse document frequency (FIDC). Phenotype-related genes were used for the evaluation of our method.

**Results:** A 7,739 × 7,739 similarity score matrix was finally obtained and the number of phenotype pairs was dramatically decreased with the increase of similarity score. Besides, the overlapping rates of phenotype-related genes were remarkably increased with the increase of similarity score between phenotypes, which supports the reliability of our method.

**Conclusion:** We anticipate our method can be applied to identifying novel therapeutic methods for complex diseases.

## Background

The rapid development of high-throughput technologies in the past decades, such as gene microarray, RNA Sequencing (RNA-Seq), and whole exome sequencing (WES), has revolutionized the evolution of biological studies. Those technologies allow the simultaneous measurement of expression values, as well as the sequence mutation and structural variation of thousands of genes, all of which greatly improve our understanding of the underlying mechanisms of complex diseases. Identification of mutated or differentially expressed genes is the beginning in the progress of the exploration of a disease in that most diseases are caused by the interplay of multiple biological processes (Menche et al., 2015). However, false positives or negatives of high-throughput experiments were one of their most common defects in the screening of valuable disease-associated information, so exploration of disease initiation and progression in other aspects is urgently needed.

Phenotypes are the observable characteristics of organisms arising from their response to stimuli *in vivo* or *in vitro* (Collier et al., 2015; Hoehndorf et al., 2015), and similar phenotypes might be induced by similar factors (van Driel et al., 2006; Kohler et al., 2008). In recent years, lots of effort has been paid toward the management of emerging scientific or clinical phenotypes in the literature. The Human Phenotype Ontology (HPO) (Kohler et al., 2014) is one of the most prevalent databases that applies standardized hierarchical terms for the description of human phenotypes through a directed acyclic graph. Based on the managed phenotypes, some algorithms were developed for their comparison and one of the approaches was to group phenotypes into clusters based on semantic similarity or other information (Groth et al., 2010; Westbury et al., 2015). In the study of Menche et al. (2015), based on the incomplete interactome of proteins, a mathematical method was proposed for the identification of disease modules and it was thought perturbations in one module could disrupt pathways of other disease modules. Groth et al. (2010) developed Phenoclustering, an online tool for the mining of cross-species phenotypes which could not only provide phenotypes clusters but phenotype descriptions, their similar Gene Ontology (GO) annotations, amino acid sequence similarity, and so on. Those methods may be of value for the study of diseases with less known or unknown pathophysiology.

Online Mendelian Inheritance in Man (OMIM) is a catalog of human phenotypes and their associated genes which is maintained by Johns Hopkins University (Amberger et al., 2011). A unique OMIM ID was assigned to every entry and different prefixes stand for different types of entries, such as "#" represents a descriptive entry, usually of a phenotype, "+" indicates the entry contains the description of a gene of known sequence and a phenotype. For phenotypes, OMIM provides us with their clinical symptoms, text description from literature which was curated by specialists, as well as their references, etc. This abundant information permits in-depth mining for the comparison between phenotypes and screening of association between phenotypes and genes. The HPO provides controlled terms for all of the phenotypic abnormalities in OMIM, which promotes the development of large-scale computational analysis and databases of the human phenome, e.g., DECIPHER (Firth et al., 2009) and ECARUCA (Vulto-van Silfhout et al., 2013), which are comprehensive databases of organized phenotype description and their potential associated chromosomal imbalance. Through applying normalized pointwise mutual information (NPMI) to co-occurrences of phenotypes and diseases in OMIM and Orphanet (Hoehndorf et al., 2013), Hoehndorf et al. (2015) developed a method for the calculation of similarity between diseases and constructed a human disease network, in which closely related diseases were clustered together. There are also some tools for the calculation of similarity between phenotypes based on semantic similarities, such as Phenomizer (Kohler et al., 2009), OWLSim (Washington et al., 2009), PhenoDigm (Smedley et al., 2013), etc., but most of them obtained the information from HPO, which might cause them to miss valuable resources in other databases. Phenomizer uses mainly the information from the directed acyclic graph of HPO, which first assigns information content (IC) to a term as the negative natural logarithm of its frequency, and then calculates the similarity between two terms as the IC of their most informative common ancestor. The similarity between two phenotypes is defined by Phenomizer as the average similarity between terms used to annotate them. OWLSim is primarily applied for cross-species phenotype comparison by using varieties of ontology-based annotation to record the affected phenotype and how it is affected. PhenoDigm is built on the top of the OWLSim algorithm, which is used for linking human diseases to model organisms for elucidating potential novel disease-gene associations, and adds zebrafish as a compared model organism in addition to mouse.

In this study, we extracted all of the phenotype entries from OMIM and retrieved their Medical Subject Headings (MeSH, a comprehensive universal controlled vocabulary for the purpose of indexing journal articles and books in the life sciences) terms within those entries. Based on those MeSH terms, PheSom (Phenotype Similarity On MeSH), a method to calculate the similarity between phenotypes, was developed. Through our method, a similarity score was assigned to phenotype-phenotype pairs and a higher score indicated higher similarity. This study would be helpful for the identification of novel candidate genes of phenotypes of interest.

## Methods

### Phenotype entries and their MeSH terms in OMIM

The entire OMIM database (http://www.ncbi.nlm.nih.gov/omim/) was downloaded, which consisted of 24,010 entries including 7,739 phenotype items. MeSH (Medical Subject Heading) terms were downloaded from the National Center for Biotechnology Information (http://www.ncbi.nlm.nih.gov/mesh/meshhome.html), and in total, 56,341 terms were obtained. Similar to HPO, MeSH terms also have a hierarchical structure with general information represented by the terms at the top level while terms at the lower level represent more detailed information. To exemplify, in Table 1, “Breast” holds more detailed information than “Body Regions”, but less detailed information than “Mammary Glands”, which indicated their different information content (IC) in describing a phenotype.

**TABLE 1:** Example of MeSH vocabularies’ level.

Vocabulary	Level	Vocabulary	Level
Body Regions	1	Extremities	2
Anatomic Landmarks	2	Amputation Stumps	3
Breast	2	Lower Extremity	3
Mammary Glands	3	Buttocks	4
Nipples	3	Foot	4

### Measuring phenotype similarity based on common MeSH terms

For each phenotype, the MeSH terms included in its OMIM entry were fetched. Briefly, MeSH terms were one-by-one searched from the text description of every OMIM entry, and the term that occurs in an entry along with those terms contained in its upper level in the MeSH hierarchical structure was assigned to the entry. The number of overlapping MeSH terms between every two phenotypes can be counted to initially measure the similarity between two phenotypes, which is referred to as VOR (vocabulary overlapping rate-based) method hereafter. Generally, two phenotypes would be more similar if they were annotated by more common terms.

### Measuring phenotype similarity based on weighted common MeSH terms

Commonly, a MeSH term occurs a different number of times (“hits”) in different phenotype entries, so it would contribute differently to those phenotypes according to the Term Frequency (TF) theory, which was first proposed by Luhn in 1957 based on the assumption that the weight of a term occurs in a document is simply proportional to the term frequency (Luhn, 1957). So, the weight of a MeSH term in a specific phenotype item could be preliminarily obtained as follows:

$$TF\left(t\right)={freq}_{tj}$$ (1)

In Eq. (1), *freq*_*tj*_ is the hits of a MeSH term *t*_*j*_ in a specific OMIM phenotype item.

While, by using the TF method alone, some common MeSH terms might be incorrectly assigned a high weight in phenotypes, such as the terms “mutation” and “patients”, and meanwhile the importance of some meaningful but low-frequency MeSH terms, such as “osteocytes” and “oogonia”, are largely overlooked. Given that point, the Inverse Document Frequency (IDF) theory, which was developed by Karen Spärck Jones based on the idea of the specificity of a term can be quantified as an inverse function of the number of documents in which it occurs (Jones, 1972), was therefore applied to overcome excessively large or small weight in TF. The weight of a MeSH term in the scenario of the total OMIM phenotype document according to IDF was calculated by the following equation:

$$IDF\left(t_{j}\right)=\log2\left(\frac{N}{DF_{j}+L}\right)$$ (2)

In Eq (2), *L* was set to 0.01 to avoid error in the condition of *DF*_*j*_ just is 0, *N* was the total number of OMIM phenotype entries, and here was 7,739, *DF*_*j*_ was the number of OMIM phenotype items which contained *t*_*j*_, i.e., Document Frequency.

By combining TF and IDF, i.e., TF-IDF, a numerical statistic that is used to reflect how important a word is to a document in a corpus, the weight of a MeSH term in a specific OMIM phenotype item in this study could be obtained via Eq (3):

$$W_{i,j}=\frac{TF\left(t_{j}\right)\times IDF\left(t_{j}\right)}{\sqrt{\sum_{j=1}^{n}{\left(TF\left(t_{j}\right)\times IDF\left(t_{j}\right)\right)}^{2}}}$$ (3)

In Eq. (3), *W*_*i,j*_ was the weight of MeSH term *t*_*j*_ in OMIM phenotype item *d*_*i*_, and *n* was the number of MeSH terms in *d*_*i*_.

According to Eq (3), a weight matrix could be obtained as follows:

$$A={\left(W_{ij}\right)}_{m\times n}=\left[\begin{array}{c} W_{11},W_{12},\ldots W_{1n}\\ W_{21},W_{22},\ldots W_{2n}\\ {\begin{array}{c} \ldots \\ W \end{array}}_{m1},{\begin{array}{c} \ldots \\ W \end{array}}_{m2},{\begin{array}{c} \ldots \\ \ldots W \end{array}}_{mn} \end{array}\right]$$ (4)

$$W_{ij}=\left\{\begin{array}{c} Eq\;3,\;if\;t_{j}\;is\;contained\;in\;d_{i}\\ 0,\;if\;t_{j}\;is\;not\;contained\;in\;d_{i} \end{array}\right.$$

In Eq. (4), *W*_*ij*_ was the weight of MeSH term *t*_*j*_ in OMIM phenotype item *d*_*i*_, *m* was the total number of OMIM phenotype records, *n* was the number of MeSH terms contained in all of the OMIM phenotype items.

In this study, cosine similarity (CS), a measurement of similarity between two vectors of an inner product space that measures the cosine of the angle between them, was used for the calculation of the similarity score between every two OMIM phenotype items. The similarity score between phenotype *d*_*i*_ *=* (*W*_*i1*_ *, W*_*i2*_ *, … … , W*_*in*_ *)* and *d*_*j*_ *=* (*W*_*j1*_ *, W*_*j2*_ *, … … , W*_*jn*_ *)* could be calculated as follows:

$$Sim\left(d_{i},d_{j}\right)={\cos\theta }_{ij}=\frac{{d_{i}d_{j}}^{T}}{\left\|d_{i}\right\|\left\|d_{j}\right\|}=\frac{\sum_{k=1}^{n}W_{ik}W_{jk}}{\sqrt{\sum_{k=1}^{n}{W_{ik}}^{2}}\sqrt{\sum_{k=1}^{n}{W_{jk}}^{2}}}$$ (5)

In Eq (5), $i,j\in \left(1,2,3\ldots m\right)$ , larger cos⁡θ represented higher similarity and the similarity score between the same phenotype record was 1. The method that takes the weight of MeSH terms into account was referred to as VW, i.e., vocabulary weighted-based method.

### Evaluation of the phenotype similarity calculation method

Similar phenotypes tend to be caused by functionally related genes or neighbors of disease genes in a network (Wu et al., 2015; Xuan et al., 2015). Here, the number of overlapping genes between the phenotype-associated genes retrieved from every OMIM phenotype item was used for the evaluation of the VOR and VW methods.

$$Overlap\left(X,Y\right)=\frac{\left|X\cap Y\right|}{\min\left(\left|X\right|,\left|Y\right|\right)}$$ (6)

In Eq (6), $\left|X\cap Y\right|$ was the number of overlapping phenotype-associated genes between two phenotype items, min (|*X*|,|*Y*|) was the number of associated genes of the phenotype from which fewer genes were fetched.

As a point of comparison, a random process was performed, in detail.

(i). Genes contained in all of the 7,739 OMIM phenotype items were fetched which obtained a total of 6,181 genes that were referred to as OMIMGene;
(ii). The number of genes contained in phenotype *d*_*i*_ and *d*_*j*_ (*i,j* = 1, 2, 3, … , m) was counted, and the same number of random genes from OMIMGene as their real associated gene number was used as the random genes of *d*_*i*_ and *d*_*j*_;
(iii). The overlapping rate of random genes of *d*_*i*_ and *d*_*j*_ was calculated through Eq (6);
(iv). Steps (i)–(iii) were repeated 100 times and the average overlapping rate between any two phenotype items was calculated.

Overlapping rates of both phenotype-associated and random genes were calculated between each phenotype item pair. Wilcoxon’s Sign Rank Test was used for the comparison between the distribution of the two types of overlapping rates.

**FIGURE 1:**
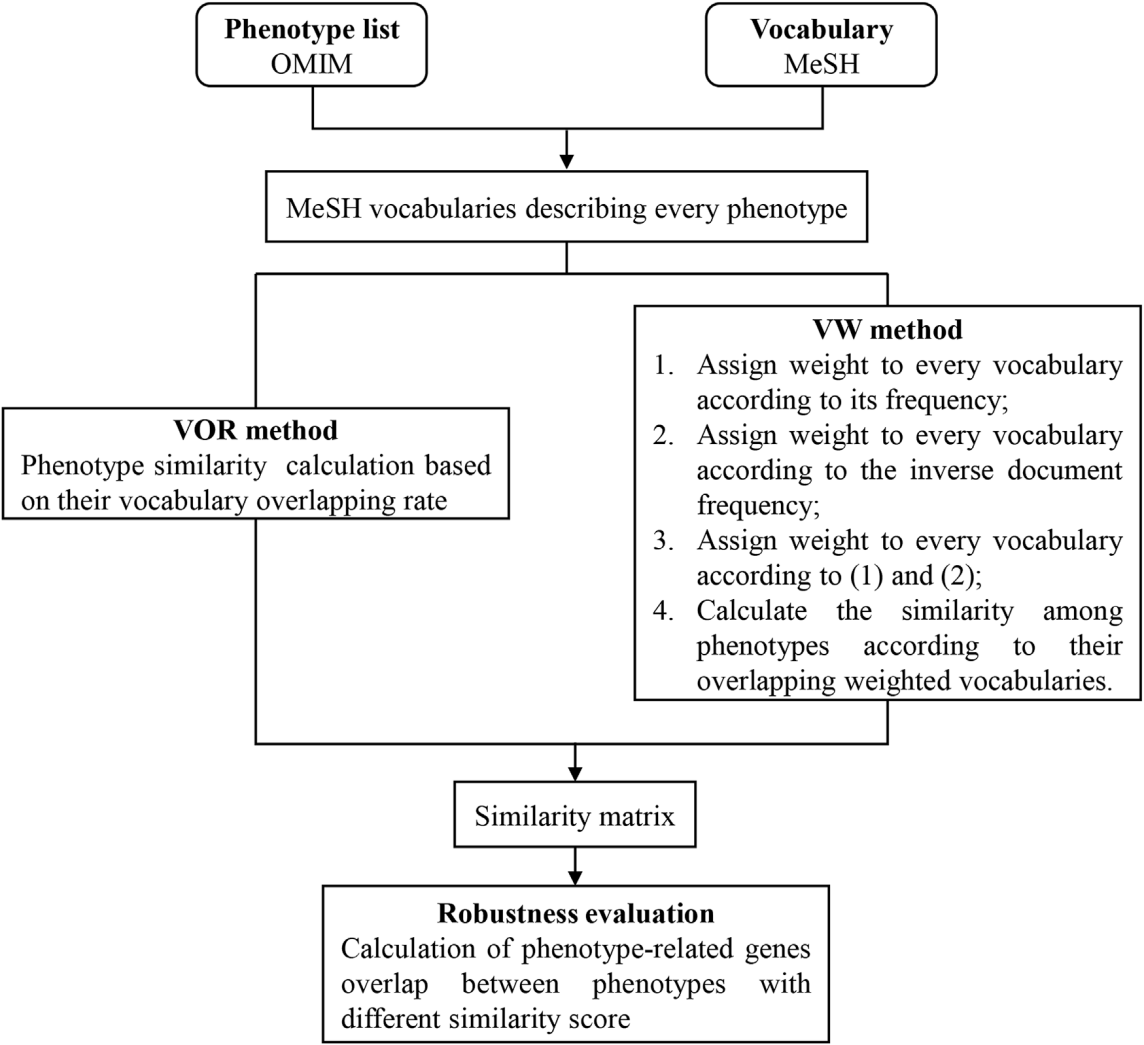
Flow chart of this study.

For the comprehensive understanding of our method, we provided a flow chart in Figure 1.

## Results

### Similarity score distribution

**FIGURE 2:**
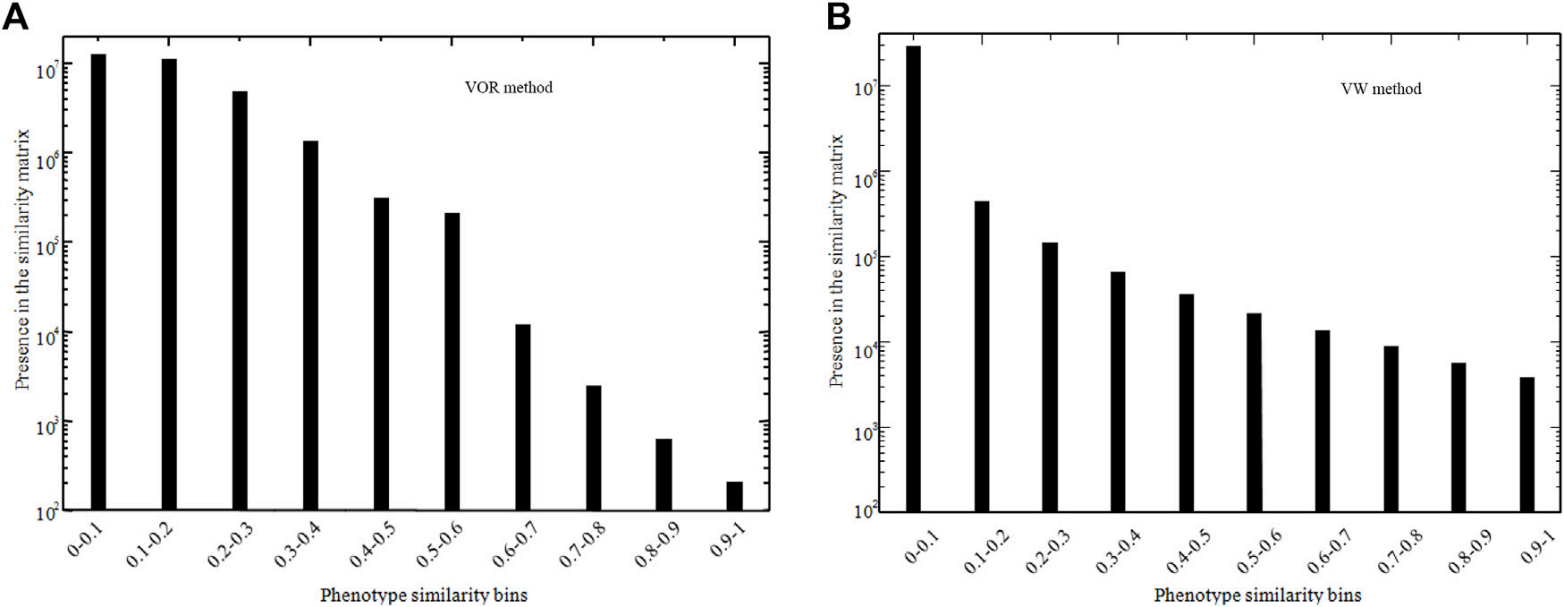
Number of phenotype pairs within different similarity score bins in VOR **(A)** and VW **(B)** methods.

A 7,739 × 7,739 similarity score matrix was obtained via the VOR and VW method in this study, and the lowest and highest score were 0 and 1, respectively. To exemplify, two 8 × 8 similarity score matrices extracted from the full matrix for the VOR and VW methods are provided in Table 2 and Table 3, respectively. Furthermore, the distribution of similarity scores across all of the 7,739 phenotype pairs was calculated based on the bin of 0.1 as shown in Figure 2. Most of the phenotype pairs had relatively low similarity scores, and only a few phenotype pairs could reach a similarity score above 0.9 through both VOR and VW methods.

**TABLE 2:** An 8 × 8 table from the similarity matrix obtained via VOR method.

Phenotype	100050	100070	100100	100200	100300	100600	100675	100700
ID								
100050	1							
100070	0.152	1						
100100	0.155	0.226	1					
100200	0.110	0.175	0.088	1				
100300	0.210	0.273	0.284	0.087	1			
100600	0.188	0.293	0.192	0.189	0.287	1		
100675	0.058	0.035	0.036	0.115	0.035	0.043	1	
100700	0.060	0.037	0.038	0.117	0.148	0.045	0.065	1

**TABLE 3:** An 8 × 8 table from the similarity matrix obtained via VW based method.

Phenotype ID	100050	100070	100100	100200	100300	100600	100675	100700
100050	1							
100070	0	1						
100100	0	0.008	1					
100200	0	0	0	1				
100300	0	0.014	0.015	0	1			
100600	0	0.017	0	0	0.005	1		
100675	0	0	0	0	0	0.043	1	
100700	0	0	0	0	0.051	0.045	0	1

### Evaluation of VOR and VW methods

**FIGURE 3:**
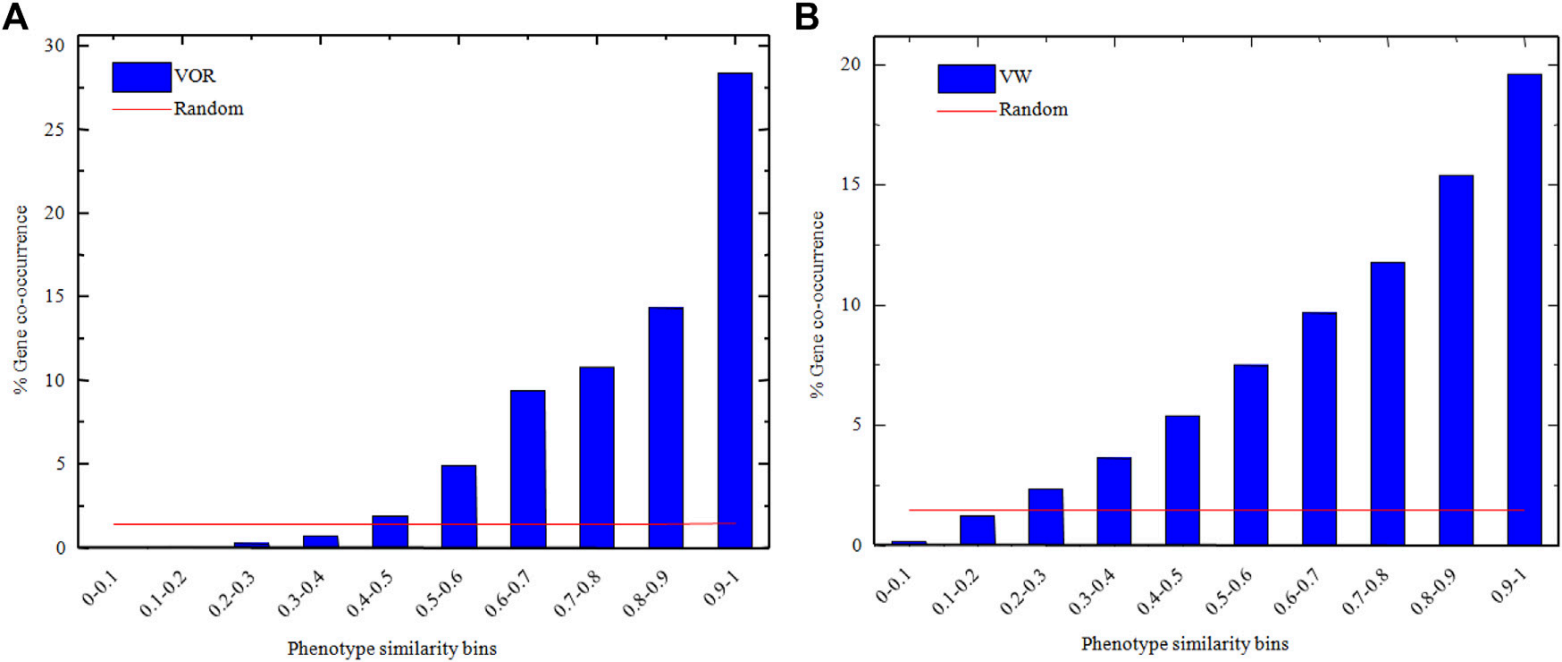
The overlapping rates of phenotypes-related genes within different similarity score bins in VOR **(A)** and VM **(B)** methods. Blue histograms and red lines represented VOR/VW methods and random gene assignment processes, respectively.

The associated genes of every phenotype were fetched from the corresponding phenotype item, and the gene overlapping rate between any two phenotype items was obtained via Eq (6). The overlapping rates within different similarity score bins in VOR and VW methods were shown in Figure 3 from which we observe that the overlapping rates were gradually increased with the increase of similarity score.

The average overlapping rates of the 100 random gene assignment processes within different similarity score bins (red line in Figure 3) were also obtained, and there was no significant difference in overlapping rates across the similarity score bins. Besides, the *p*-value of Wilcoxon’s Sign Rank Test (Wilcoxon, 1946) between the overlapping rates obtained through the random gene assignment process and the VOR method, as well as the random gene assignment process and the VW method were all less than 0.05, which indicated that the overlapping rates were significantly different between our methods and the random gene assignment process.

### Comparisons between our own phenotype comparison methods and three others

**FIGURE 4:**
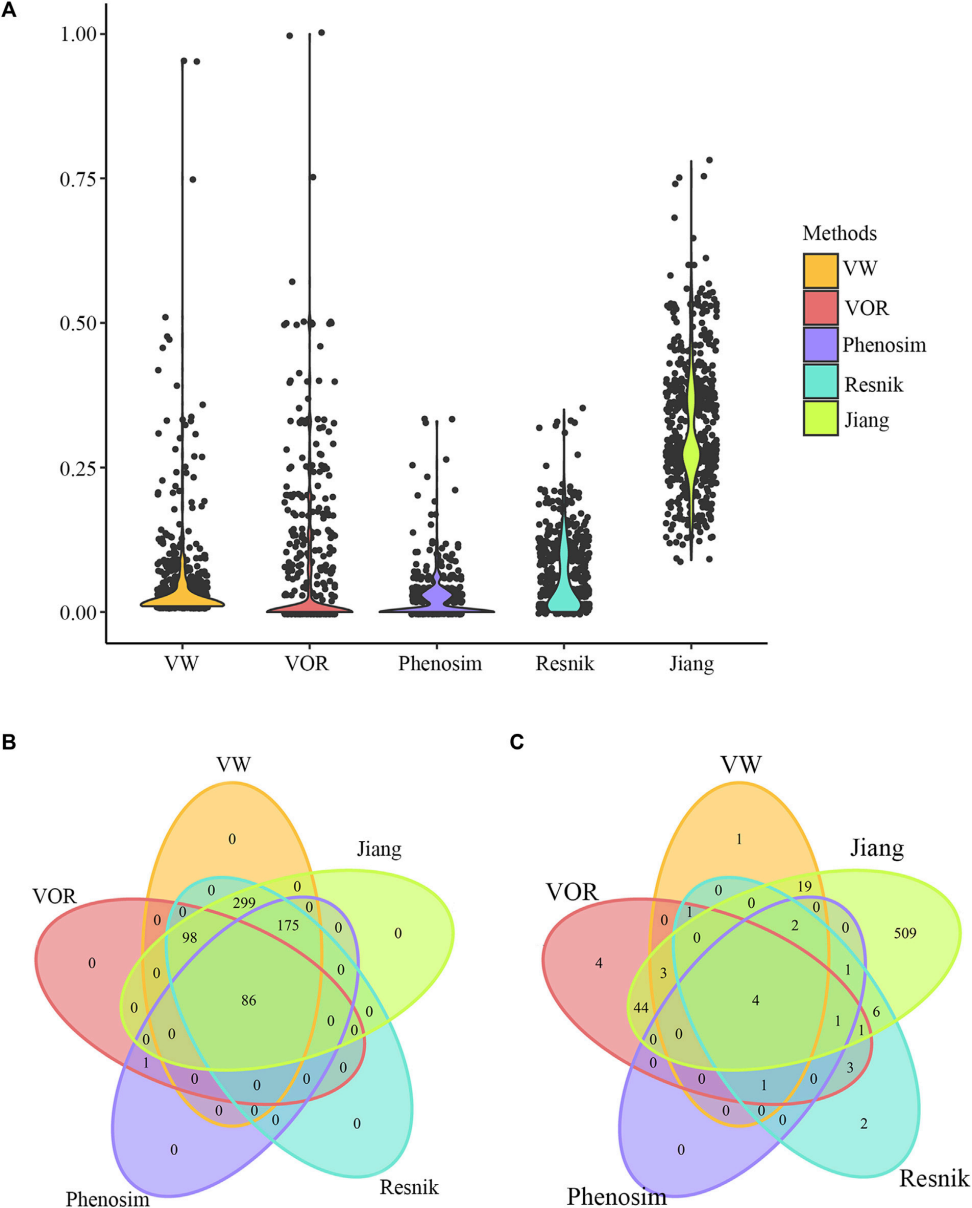
Phenotype comparison results of breast cancer. **(A)** Distribution of similarity scores calculated by VW, VOR, Phenosim, Renisk, et al., and Jiang and Conrath (1997), between breast cancer and the 658 phenotypes that had similarity scores greater than 0 obtained through the VW method. **(B)** Venn diagram depicting overlaps of phenotypes that had similarity scores with breast cancer greater than 0 in all the five methods. **(C)** Venn diagram depicting overlaps of phenotypes that had similarity scores with breast cancer greater than 0.2 in all the five methods.

We used breast cancer as a specific example to compare the performance of our methods and three other common phenotype comparison methods, i.e., Phenosim, Jiang and Conrath (1997) and Resnik (1995). Similarity scores between breast cancer and the remaining phenotypes were calculated by the VW method, and a total of 658 phenotypes had similarity scores greater than 0. Similarity scores between breast cancer and each of those 658 phenotypes were then calculated by VOR, Phenosim, Jiang and Conrath (1997) and Resnik (1995). Jiang and Conrath (1997) and Resnik (1995) mainly used the combination of information content and lexical taxonomy to evaluate semantic similarity. Phenosim was primarily designed for simulating phenotypes, by which phenotypic similarity could also be obtained based on the genetic and epidemiology information. As a result, the distribution of similarity scores between breast cancer and other phenotypes calculated by all five methods were comparable except for Jiang and Conrath (1997), which obtained relatively higher similarity scores than the other four methods as shown in Figure 4A. Besides, there were 86 (Figure 4B; Supplementary Table S1) and 4 phenotypes (Figure 4C; Table 4) that had similarity scores greater than 0 and 0.2 in the results of all five methods, respectively. Those data indicate that our methods have reliable performance in calculating phenotype similarity and should be complementary with other methods.

**TABLE 4:** The 4 overlapping phenotypes that had similarity score with breast cancer greater than 0.2 in all the five methods.

Phenotype title	VOR	VW	Phenosim	Resnik	Jiang
Li-fraumeni syndrome	0.333	0.253	0.205	0.306	0.528
Ovarian cancer	0.318	0.313	0.263	0.321	0.649
Breast-ovarian cancer, familial, susceptibility to, 1	0.455	0.954	0.329	0.326	0.748
Breast-ovarian cancer, familial, susceptibility to, 2	0.500	0.950	0.329	0.326	0.748

### Phenotypes similar to nicotine addiction

Nicotine addiction is one of the most common substance-abuse diseases whose development is associated with many genes and pathways (De Palma et al., 2010; Quik et al., 2011; Durazzo et al., 2015). In our previous study, 220 genes were identified as the optimal nicotine addiction-related genes through a multi-source-based approach (Liu X. et al., 2015). Besides, biochemical pathways related to neurodevelopment, the immune system, and metabolism were found to be enriched in those 220 genes in another study of ours (Liu et al., 2015b). Here, based on VOR and VW methods, some phenotypes similar to nicotine addiction were obtained and the top 5 phenotypes according to the similarity score were provided in Table 5, all of which were closely associated with substance abuse and immune deficiency or tobacco-use related lung cancer. Additionally, a total of 27 nicotine addiction-related genes (Supplementary Table S2) were fetched from its OMIM phenotype item, and 19 out of which were found to be overlapped with the 220 prioritized genes in our previous study (Liu et al., 2015b). A total of 29 and 15 phenotype-related genes were fetched for the top 5 phenotypes obtained through VOR and VW methods, and there were 11 and 9 overlapping genes between the 29 and 15 phenotype-related genes and the 220 previously prioritized genes (Liu et al., 2015b), respectively. The remaining genes of those similar phenotypes might provide novel candidate genes for nicotine addiction.

**TABLE 5:** The top 5 similar phenotypes of nicotine addiction obtained by VOR and VM methods.

Phenotype ID	Phenotype title	Similar score
VOR method		
183100	spinocerebellar atrophy with pupillary paralysis	0.422
610065	systemic lupus erythematosus, susceptibility to, 7	0.422
610066	systemic lupus erythematosus, susceptibility to, 8	0.422
612253	systemic lupus erythematosus, susceptibility to, 11	0.421
103780	alcohol dependence;alcoholism	0.416
VW method		
611003	Smoking as a quantitative trait locus 1	0.685
606581	Polysubstance abuse, susceptibility to; psab; drug addiction, susceptibility to	0.449
611004	Smoking as a quantitative trait locus 2	0.441
613778	Macular degeneration, age-related, 8	0.368
608935	Lung cancer susceptibility 1	0.304

## Discussion

In the post-genomic era, screening of candidate genes becomes a more and more prevalent method for the study of complex genetic diseases (Botstein and Risch, 2003; McCarthy et al., 2003; Oti and Brunner, 2007) and it is important for the improvement of medical care (Luo and Liang, 2015). Lots of methods have been proposed for this purpose, including Genome-wide association studies (GWAS) (Lewis et al., 2011; Martelle et al., 2016; Smith et al., 2016), whole exome sequencing (Friedman et al., 2014; Butler et al., 2015; Chapman et al., 2015; Bruse et al., 2016), as well as network-based methods (Yao et al., 2011; Luo and Liang, 2015), etc. Some disease-gene/protein, as well as gene-protein relationships, were uncovered, while little effort has been applied to the relationships at the phenotype level, which would be of benefit for the biological interpretation of complex diseases with similar phenotypes that might be caused by functionally related genes. Here, we developed a novel method that could robustly estimate the similarity among phenotypes deposited in the OMIM database. This study finally obtained a matrix containing pairwise similarities among 7,739 phenotypes and there are more overlaps between phenotype-related genes among phenotypes that exhibit higher similarity than those exhibit lower similarity.

Most of the human Mendelian syndromes have been deposited in the OMIM database and described in detail, while the lack of a controlled term to consistently annotate them has limited the development of computational tools at the phenotype level in the past. For this purpose, HPO was developed for the improvement of annotation of phenotypes in OMIM with controlled terms in the form of a directed acyclic graph, which could also be used to calculate phenotypic similarities between diseases (Robinson et al., 2008). Some methods or tools have been proposed for the measurement of phenotypic similarity of diseases based on HPO. Through semantic similarity metrics and taking the interrelationships between terms in HPO into account, Kohler et al. (2009) developed a web-based application for the human Mendelian disorders, from which a similarity score and *p*-value could be obtained for the rank of similar diseases. Deng et al. (2015) even developed an HPO-based R package, HPOSim, for the calculation of phenotypic similarity through seven commonly used semantic similarity measures: Resnik measure (Resnik, 1995), Lin measure (Lin, 1998), Jiang-Conrath measure (Jiang and Conrath, 1997), relevance measure (Schlicker et al., 2006), information coefficient measure (Li et al., 2010), graph IC measure (Pesquita et al., 2007) and Wang measure (Wang et al., 2007). Besides, hypergeometric enrichment analysis and network ontology analysis could also be conducted via HPOSim While, few studies employed the MeSH terms on the calculation of phenotype similarity, which is a valuable medical controlled vocabulary similar to HPO.

In this study, the PheSom (VOR and VW) method was developed for the comparison of phenotypes in OMIM, and two 7,739 × 7,739 similarity score matrices were obtained. The overlapping rates of phenotypes-related genes in different similarity score bins indicated our method is reliable in identifying similar phenotypes which would be helpful in the collection of novel candidate genes for complex diseases. Sarkar IN (Sarkar, 2012) proposed a vector space model-based method, which implicates two vectors including gene vector, i.e., genes that are associated with queried genes that are directly related to a disease retrieved through BLAST+ from GeneBank, and a disease vector that is the quantification of relative relationships between candidate diseases and the related genes, to identify genetically related diseases. Resemblances indeed exist between Sarkar’s and this study, such as vector-based representation for phenotype and cosine similarity for quantification of the relationship between phenotypes. However, differences in the materials used in the two studies, i.e., genetic information for diseases in Sarkar’s study and MeSH term annotation for diseases in this study, differentiate the two studies and suggest they may complement each other.

The number of phenotype pairs decreased with the increase of similarity score in both of the two methods and there were only 0.501% and 1.048% out of all phenotype pairs with a similarity score >0.6 in VOR and VW methods, respectively. Our results were consistent with the study of Driel et al., which compared the similarity between phenotypes in OMIM based on the text mining analysis of MeSH terms inside the phenotypes records (van Driel et al., 2006). This might indicate the low similarity between most of the phenotypes. While, we should pay attention to some conditions which would influence the calculation of the similarity score, such as if *t*_*j*_, a MeSH term, is important for the description of *d*_*i*_, an OMIM phenotype item, but the hits of *t*_*j*_ are low in *d*_*i*_, its weight in *d*_*i*_ would be lower than expected according to Eq. (2), and the similarity score might become lower between *d*_*i*_ and the other phenotypes contained more *t*_*j*_. Xue et al. also presented a study for estimating phenotype similarity based on HPO terms by incorporating not only HPO structure but terms’ definition. Several similarities exist between Xue’s and our study, e.g., TF-IDF method, and cosine similarity, while phenotype annotations used in our study are standardized MeSH terms fetched from every OMIM entry, which were manually reviewed and should be reliable and comprehensive.

For nicotine addiction, the two methods obtained some phenotypes with high similarity scores. Table 5 indicated that the similar phenotypes obtained through VOR and VW were mainly involved in immune- and substance-abuse-related processes respectively. Nicotine addiction is a substance-abuse disease that could also reduce the immune response (Schumacher, 2013; Alving et al., 2014; Liu et al., 2015b; Mishra et al., 2015) and pose similar mechanisms to the addiction to other substances, such as drugs (Motlagh et al., 2016; van Wel et al., 2016). Besides, compared with the prioritized genes of nicotine addiction obtained in our previous study, many overlapping genes were identified and the overlap rates were 38% and 60% in VOR and VW methods respectively, which indicated the reliability of our two methods and VW might outperform than VOR. Some discrepancies also existed between nicotine addiction-related genes that were prioritized in our previous study and genes that were contained in the top 5 most similar phenotype entries. An example is FAAH, which is recorded as a related gene for the second most similar phenotype, i.e., “POLYSUBSTANCE ABUSE, SUSCEPTIBILITY TO; PSAB”, for nicotine addiction by VW method, was not prioritized by our previous method. However, the association between FAAH and nicotine addiction is supported by some other studies (Simonnet et al., 2017; Pavon et al., 2018). STAT4 represents another gene that is included in the top five most similar phenotype items but was not identified by our previous study. STAT4 is a transcriptional factor encoding gene that is phenotypically associated with immune-related diseases, such as systemic lupus erythematosus and rheumatoid arthritis (Salmaninejad et al., 2017; Ebrahimiyan et al., 2019). Nicotine addiction has been previously reported to be immune dysregulation-related (Wang et al., 2011; Liu et al., 2015c), so STAT4 might serve as a potential candidate for nicotine addiction.

This study provides the most comprehensive OMIM-based comparisons among different phenotypes so far. Our method directly quantifies the similarity among phenotypes, which would be helpful for the drug repurposing in the scenario of the existence of well-known drugs for one phenotype but the candidate drug is lacking in highly similar phenotypes. Besides, this study should also be helpful for identifying novel candidate genes for some diseases in similar phenotypes that might share causal genes. Limitations of this study do exist, such as the similarity matrix should be manually updated with the constant expansion of vocabulary describing the physiological or pathological states. Additionally, it would be better to add the laboratory-based validation for the novel related genes of specific diseases identified through our method.

## Conclusion

In conclusion, we developed two methods for the calculation of similarity scores between phenotypes in OMIM through the semantic similarity of MeSH terms. The overlapping rates of phenotype-related genes in different similarity score bins indicate the reliability of our methods and suggest they would be helpful for the identification of novel candidate genes of complex genetic diseases.

## Data Availability

Publicly available datasets were analyzed in this study. This data can be found here: https://www.omim.org/.

## Abbreviations

RNA-Seq, RNA Sequencing; WES, whole exome sequencing; HPO, Human Phenotype Ontologies; GO, Gene Ontology; OMIM, Online Mendelian Inheritance in Man; MeSH, Medical Subject Headings; PheSom, Phenotype Similarity On MeSH; NPMI, normalized pointwise mutual information; IC, information content; TF, Term Frequency; IDF, Inverse Document Frequency; CS, cosine similarity; VOR, vocabulary overlapping rate-based; VW, vocabulary weighted; GWAS, Genome-wide association study.
